# Personality and cognition across the lifespan: investigating their interplay and age-related differences

**DOI:** 10.3389/fpsyt.2026.1925413

**Published:** 2026-10-07

**Authors:** Mats Najström, Ove Almkvist, Maria Lindau

**Affiliations:** 1 Department of Psychology, Stockholm University, Stockholm, Sweden; 2 Department of Neurobiology, Care Sciences and Society, Karolinska Institutet, Stockholm, Sweden

**Keywords:** aging, cognitive functioning, facet-level analysis, personality traits, personality-cognition interaction

## Abstract

**Introduction:**

Personality-cognition associations may vary across adulthood, but findings are inconsistent. The present study examined associations between personality traits and cognitive functioning within younger and older adults and explored whether these associations differed across age groups.

**Methods:**

A cross-sectional design was used. Participants were 191 adults divided into younger (N = 99; 19–49 years) and older (N = 92; 50–87 years) groups. Cognitive functioning was assessed with the Wechsler Adult Intelligence Scale-IV, and personality with the Swedish Universities Scales of Personality. Between-group comparisons, covariate-adjusted hierarchical regressions, and exploratory facet-level and personality-configuration analyses were conducted.

**Results:**

Younger adults obtained higher age-normed scores than older adults on Full-Scale IQ, Working Memory, and Processing Speed and reported higher Neuroticism. In younger adults, higher Extraversion was independently associated with lower Full-Scale IQ after adjustment for chronological age, sex, education, Neuroticism, and Aggressiveness. A nominal negative association between Extraversion and Verbal Comprehension did not remain significant after Holm correction. Exploratory facet-level analyses yielded significant overall models for Full-Scale IQ and Verbal Comprehension, with Impulsivity uniquely associated only with Verbal Comprehension. In older adults, no significant one-dimensional personality-cognition associations were observed. Exploratory analyses indicated that a high-Extraversion/low-Neuroticism configuration was associated with better Working Memory and Processing Speed; facet-level characterization implicated Impulsivity, Adventure Seeking, Stress Susceptibility, and Psychic Trait Anxiety.

**Discussion:**

The findings suggest a direct association between Extraversion and global cognitive functioning in younger adults, whereas exploratory findings in older adults suggest more configurational personality-cognition patterns. Replication in larger, independent samples is warranted.

## Introduction

Cognition and personality are two fundamental aspects of human behavior that have been extensively studied, with a substantial body of research examining their interaction (1–3). Although personality traits are known to relate to cognitive abilities, particularly within specific cognitive domains, findings remain inconsistent. Some studies report strong associations, whereas others identify weak or negligible relationships (4–6). Furthermore, the role of age in shaping personality–cognition associations is insufficiently explored, and existing studies addressing this interaction across the lifespan have produced contradictory results (7, 8). As both cognition and personality show age-related stability and change, a lifespan perspective is essential to clarify how these constructs interact across different age groups.

### Cognition

The structure of cognition has been debated for over a century, resulting in several theoretical frameworks (9, 10). Contemporary models commonly emphasize a hierarchical organization of cognitive abilities, with broad domains encompassing more specific functions (11). Cognitive abilities are frequently assessed using the Wechsler Adult Intelligence Scale (WAIS) (12), which operationalizes constructs derived from the Cattell–Horn–Carroll (CHC) model. This framework distinguishes between fluid intelligence, reflecting reasoning and problem-solving in novel situations, and crystallized intelligence, representing knowledge acquired through education and experience (12, 13).

The WAIS provides a comprehensive assessment of cognitive functioning across multiple domains and highlights cognition as a multifaceted construct rather than a unitary ability (14). Earlier research described cognitive functioning as relatively stable throughout adulthood (15, 16), but more recent findings indicate age-related increases in semantic knowledge alongside declines in executive functioning, memory, and visuospatial abilities (17–20).

### Personality

Personality is commonly conceptualized as a configuration of traits rather than a single dimension (21, 22). Given its multidimensional nature, numerous self-report instruments have been developed to assess distinct personality characteristics (23). Among the most influential frameworks is the Five-Factor Model (FFM), which comprises Openness, Conscientiousness, Extraversion, Agreeableness, and Neuroticism (24–26). Widely used instruments include the Minnesota Multiphasic Personality Inventory (27), the NEO Personality Inventory (28), and the Swedish Universities Scales of Personality (SSP), which is applied in the present study (29). The relationship between FFM and SSP is illustrated by Agreeableness in FFM versus the facet Social Desirability in SSP (30) and Conscientiousness in FFM versus the facet Impulsiveness in SSP (23). These results indicate that facets of SSP may be relevant for factors of FFM that was not directly assessed by SSP.

Earlier theories proposed that personality stabilizes after early adulthood (31), but contemporary research increasingly characterizes personality as a dynamic system that remains responsive to socialization, maturation, and life experiences across the lifespan (32–34). Empirical evidence suggests ongoing personality plasticity, including age-related trends toward reduced Neuroticism and increased Agreeableness (33, 35). Emerging research further indicates that personality traits can be modified through non-clinical psychological interventions (36–38). For example, coaching and psychotherapy-based approaches have been associated with changes in traits such as Conscientiousness, Extraversion, and Neuroticism (39–41). However, methodological challenges remain, and substantial evidence also supports relative stability in personality traits across adulthood (42–44).

Together, these findings suggest that personality is neither entirely fixed nor entirely malleable, underscoring the importance of examining personality–cognition relationships within a lifespan framework.

### Cognition and personality

The interdependence between cognition and personality has received substantial empirical attention, with evidence suggesting meaningful interactions between the two constructs (3). Research has mainly focused on cross-sectional associations between cognitive abilities and personality traits, although findings remain heterogeneous. Among the most consistently examined traits are Neuroticism, Extraversion, and Openness.

Neuroticism is often associated with poorer cognitive performance, particularly in memory, visuospatial ability, and executive functioning (5, 45). Several studies report negative associations between Neuroticism and a range of cognitive domains, including global cognition and verbal fluency (46–48). However, other studies have failed to identify significant relationships between Neuroticism and cognitive performance (4, 49), and some findings suggest indirect rather than direct effects, mediated by factors such as perceived stress (48). Overall, Neuroticism has been suggested as a potential risk factor for cognitive decline and dementia, but the strength and consistency of this association vary across studies and cognitive domains (6).

Findings regarding Extraversion and cognition are similarly mixed. Extraversion has been linked to enhanced creativity and memory but poorer performance in spatial orientation, reasoning, and verbal ability (45, 50). While some studies report negative associations with reasoning (51), others demonstrate positive or null associations depending on the cognitive domain assessed (4, 5, 46).

In contrast, Openness has shown the most consistent positive association with cognitive functioning in studies based on the Five-Factor Model (7, 52–54). Associations between cognition and the remaining Five-Factor Model traits are generally weak or inconsistent. Variability across studies may partly reflect differences in age composition, measurement instruments, and analytical approaches, including the handling of moderators and mediators (55).

To address these inconsistencies, increasing attention has been directed toward facet-level analyses. Evidence suggests that narrower personality facets provide greater explanatory precision than global traits and show distinct associations with different outcomes (56, 57). For example, different facets of Extraversion have been shown to relate uniquely to health and life satisfaction (58).

Beyond single traits, personality characteristics are often understood as interacting configurations (21, 22). Emerging research indicates that personality traits may jointly influence cognitive performance, suggesting that associations observed for one trait may depend on the level of another. For instance, the relationship between Neuroticism and functional outcomes has been shown to be moderated by Conscientiousness (59, 60), and low Emotional Stability or Openness appears to drive lower cognitive performance (61). These findings highlight the importance of a multidimensional approach that accounts for interactions among traits.

### Cognition, personality and age

Both cognition and personality undergo changes across the lifespan, making age a plausible moderator of their interrelationship. Longitudinal evidence suggests covariation between changes in cognitive functioning and personality traits over time (8). Individuals exhibiting declines, stability, or increases in personality traits have shown differing patterns of cognitive aging, including changes in executive functioning and episodic memory.

Age-related differences in personality–cognition associations have been observed in some studies. For example, higher Neuroticism was associated with lower reasoning ability in younger adults but not in older adults, while specific Extraversion facets were linked to better verbal fluency in older individuals (52). These findings suggest that facet-level associations may vary with age and that relationships between personality and cognition may be non-linear (57, 58).

However, other large cross-sectional studies have reported relatively stable personality–cognition associations across adulthood, with only weak or inconsistent moderation by age (3, 7). In particular, findings based on Five-Factor Model measures suggest that the positive association between Openness and cognitive functioning persists across a broad age range (7, 52–54).

Previous research suggests that personality traits and cognitive functioning are associated across adulthood, although findings regarding the consistency and strength of these associations have been mixed. Furthermore, comparatively few studies have examined these associations across multiple cognitive domains in both younger and older adults using the same methodological framework. Given the cross-sectional nature of most available evidence, including the present study, observed differences between age groups should be interpreted as group differences rather than developmental changes over time.

### Aim of the study

Against this background, the primary objective of this study was to examine associations between personality traits and cognitive functioning across multiple cognitive domains within younger and older adult groups. A secondary objective was to explore whether the pattern and strength of these associations differed between groups. The research question was: How are personality traits associated with cognitive functioning within younger and older adults, and do the patterns of association differ between age groups? We hypothesized that personality traits would be associated with cognitive functioning and that the pattern of these associations would differ between younger and older adults. Given the inconsistent findings in previous research, analyses examining the specific nature, strength, and structure of these associations were considered exploratory. In interpreting age-group differences, potential influences of factors such as educational attainment and sex should also be considered.

## Methods

### Participants

Participants volunteered to be assessed by psychology students as part of the students’ required training in cognitive and personality assessment in the Master program at the Department of Psychology, Stockholm University. Participants comprised younger adults (*N* = 99, 44.44% females) and older adults (*N* = 92, 57.6% females). The age distribution was bimodal; M = 29.7, SD = 7.5 (range 19–49) in the younger group and M = 67.2, SD = 8.7 (range 50–87) in the older group. Given the bimodal age distribution, age group was treated as a categorical variable in the between-group analyses. Within each age group, chronological age was retained as a continuous covariate in the hierarchical regression analyses. Accordingly, analyses focused on comparisons between age groups and within-group associations rather than modelling age as a continuous moderator. Information on years of education was missing for 22 participants. For the remaining 169 participants, no significant group difference in years of education was observed, *t*(167) = -1.29, p = .199. Furthermore, sex distribution did not differ significantly between groups, χ²(1, N = 191) = 3.29, p = .070. To ensure objectivity, a close relationship between the student and the recruited participants was to be avoided. Nevertheless, the sample must be considered a biased convenience sample. The inclusion criteria required participants to report good subjective health and to belong to an age group either similar to or older than that of the student. Each student was tasked with recruiting both a younger and an older adult. During the screening interview, participants were asked about current and previous medical and psychiatric conditions as well as medication use. Individuals reporting conditions considered likely to interfere with study participation or cognitive functioning were not included in the study. In addition to the WAIS-IV assessment, participants completed several other cognitive tests, including measures of episodic memory, attention, and executive functioning, and no findings indicating clinically significant cognitive impairment were observed.

### Procedure

The students were responsible for recruiting study participants. Cognitive testing was conducted using the Scandinavian version of the WAIS-IV (12), while personality was assessed using the SSP (29). Prior to data collection, all students had received formal training in the administration and scoring of both instruments as part of their Master’s-level education in psychology. Testing was conducted in a quiet room, either in the participant’s home or at the university, and the complete procedure took approximately 3 to 4 hours, including breaks.

Following administration, the WAIS-IV and SSP protocols were scored according to the respective manuals and reviewed by the supervising psychologist. The verified scores were entered into a database structured with one row per participant and separate variables for demographic characteristics, WAIS-IV indices, SSP facets, and higher-order personality factors. The data were checked for completeness and consistency before being prepared for statistical analysis in SPSS. Participants with incomplete WAIS-IV or SSP assessments were excluded from the relevant analyses. Missing data on years of education were handled using complete-case analysis in the covariate-adjusted regression models. Participants were also excluded if their test results indicated possible cognitive or personality-related impairment.

Once the testing and assessment report had been reviewed and approved by the supervisor, the student provided individualized feedback to the participant in person. The feedback was tailored to the participant’s specific interests and concerns. All procedures were conducted under the supervision of experienced licensed psychologists at Stockholm University.

### Instruments

The WAIS battery was administered in its entirety and scored according to standardized procedures described in the Scandinavian version of WAIS-IV (12). The ten subtests were summarized into four indices: Verbal Comprehension Index (VCI), Perceptual Reasoning Index (PRI), Working Memory Index (WMI), and Processing Speed Index (PSI) along with a summary index of global cognition, the Full-Scale Intelligence Quotient (FSIQ). Raw subtest scores were converted to age-normed scaled scores and subsequently combined into age-normed index scores and Full-Scale IQ according to the Scandinavian WAIS-IV manual. Standard WAIS-IV age-normed scores were used throughout the analyses; no W-scores were used.

The SSP is a self-rating scale that measures stable personality traits based on existing theories concerning personality constructs and separate biological theories related to psychiatric disorders rather than the full spectrum of personality. In addition, the SSP was developed to improve upon the psychometric properties of the Karolinska Scales of Personality (KSP) (62) and a standardization study was carried out based on a random sample of the Swedish population (20 ± 64 years of age) (29). The SSP includes 91 items that participants answer on a Likert scale ranging from 1 to 4 (1 = do not agree at all, 4 = agree totally). This instrument consists of 13 subscales divided into three overall factors: 1) Neuroticism, 2) Aggressiveness, and 3) Extraversion. The internal consistency of the personality trait measures in the present sample, as indicated by Cronbach’s alpha, was acceptable: Extraversion (α = 0.73), Neuroticism (α = 0.82), and Aggressiveness (α = 0.71).

### Statistical analyses

Statistical analyses were conducted using SPSS version 28.0.1.0. Given the bimodal age distribution, analyses distinguished between between-group comparisons (younger vs. older adults) and within-group associations. Group differences were examined using independent-samples t-tests. Within-group associations between personality and cognition were examined using hierarchical regression analyses, with chronological age, sex, and years of education entered in the first step and Extraversion, Neuroticism, and Aggressiveness entered in the second step. Regression analyses were based on complete cases because years of education was missing for some participants. The analytical sample size is therefore reported for the adjusted models. Full-Scale IQ was analyzed separately as a global measure of cognitive functioning, whereas VCI, PRI, WMI, and PSI were treated as a family of domain-specific cognitive outcomes. Holm’s sequential procedure was applied across the four domain-specific outcomes separately for each personality trait. Both unadjusted and Holm-adjusted results are reported. Subsequent unadjusted facet-level regression, personality-configuration, ANOVA, and MANOVA analyses were conducted to characterize the observed patterns and were considered exploratory.

## Results

**Tables 1** and **2** summarize the descriptive statistics for the demographic variables, cognitive measures and personality traits for the younger and older groups.

**Table 1:** Descriptive statistics of the young group.

Variable	N	M	SD	Min	Max
Age	99	29.70	7.51	19	49
Cognitive measures					
VCI	99	117.91	13.45	81	145
PRI	99	117.19	14.21	82	144
AI	99	113.71	15.63	85	150
PSI	99	108.91	12.98	76	136
FSIQ	99	116.72	12.10	83	141
Personality traits*					
Neuroticism	99	255.33	34.93	184.20	350.49
Extraversion	99	150.08	16.19	114.82	190.38
Aggressiveness	99	243.94	26.53	187.18	316.74

*T-values.

**Table 2:** Descriptive statistics of the older group.

Variable	N	M	SD	Min	Max
Age	91	67.18	8.76	50	87
Cognitive measures					
VCI	91	118.16	11.79	93	143
PRI	91	111.45	13.11	74	134
WMI	91	109.24	12.74	76	139
PSI	91	107.01	11.34	71	134
FSIQ	91	113.46	9.44	85	130
Personality traits*					
Neuroticism	91	242.65	32.82	189.96	326.97
Extraversion	88	151.28	16.39	112.67	188.04
Aggressiveness	88	239.02	25.13	185.28	319.39

*T-values.

### Between-group differences in cognition and personality

To examine overall differences between age groups, younger and older adults were compared on cognitive and personality measures using independent samples t-tests. The younger group performed significantly better than the older group on several cognitive measures, including FSIQ [*t*(188) = -2.076, *p* <.05], WMI [*t*(188) = -2.165, *p* <.05], and PSI [*t*(188) = -2.896, *p* <.01]. There was also a significant difference between the two age groups in Neuroticism [*t*(188) = -2.572, *p* <.05]; the younger group exhibited higher levels of Neuroticism.

### Within-group associations between personality and cognition

To examine personality-cognition associations while accounting for demographic variation within the age groups, hierarchical regression analyses were conducted separately for younger and older adults. Chronological age, sex, and years of education were entered in the first step, followed by Extraversion, Neuroticism, and Aggressiveness in the second step. Both unadjusted and Holm-adjusted results are reported.

Among younger adults with complete data (*N* = 78), demographic variables explained 19.4% of the variance in FSIQ, *R²* = .194, *F*(3, 74) = 5.94, *p* <.001. Adding the personality traits significantly improved the model, explaining an additional 13.7% of the variance, Δ*R²* = .137, *F*_change_(3, 71) = 4.85, *p* = .004 (*f²* = .20). In the final model, higher Extraversion was uniquely associated with lower FSIQ (B = -1.59, SE = 0.62, β = -.29, *p* = .016), whereas Neuroticism and Aggressiveness were not significant predictors.

For VCI, demographic variables explained 16.6% of the variance, *R²* = .166, *F*(3, 74) = 4.91, *p* = .004. The addition of the personality traits did not significantly improve the model, ΔR² = .060, *F*_change_(3, 71) = 1.84, *p* = .147. However, higher Extraversion was uniquely associated with lower VCI (B = -1.44, SE = 0.63, β = -.26, unadjusted *p* = .025). No significant personality-cognition associations were observed for PRI, WMI, or PSI.

Because the four WAIS-IV index scores (VCI, PRI, WMI, and PSI) were treated as a family of domain-specific cognitive outcomes, Holm’s sequential correction was applied across these analyses. The association between Extraversion and VCI did not remain significant after correction for multiple testing (Holm-adjusted *p* = .100). Consequently, the negative association between Extraversion and FSIQ, analyzed separately as the global cognitive outcome, was the only robust trait-level personality-cognition association in the younger group. Nevertheless, the nominally significant association between Extraversion and VCI (unadjusted *p* = .025, Holm-adjusted *p* = .100) was considered sufficient to motivate the subsequent exploratory facet-level analyses.

Among the older adults with complete data (*N* = 91), the same analytical procedure was applied. Across all cognitive outcomes (FSIQ, VCI, PRI, WMI, and PSI), the addition of the personality traits did not significantly improve any model, and none of the three personality traits showed a significant unique association with cognitive performance. Thus, no one-dimensional personality-cognition associations were observed in the older group.

### Facet-level regression analyses – younger group

To further explore the observed association between Extraversion and cognitive functioning in the younger group, separate unadjusted exploratory facet-level regression analyses were conducted. Extraversion was decomposed into the facets Impulsivity, Adventure Seeking, and Detachment. FSIQ was selected as the primary outcome because it remained significantly associated with Extraversion after adjustment for demographic variables and the remaining personality traits. VCI was also examined because a nominally significant association with Extraversion was observed in the unadjusted analyses.

VIF values ranged from 1.16 to 1.28, indicating no problematic multicollinearity among the facets. Multiple regression analyses identified two significant models. For FSIQ, the model explained 7.9% of the variance, *F*(3, 95) = 2.70, *p* <.05, although none of the individual facets made a significant unique contribution. For VCI, the model explained 8.8% of the variance, *F*(3, 95) = 3.07, *p* <.05, with Impulsivity emerging as the only significant predictor.

These findings illustrate the value of facet-level analyses in clarifying within-group associations between personality and cognitive performance. **Table 3** presents the regression coefficients for the predictor variables included in the model.

**Table 3:** Regression analysis of VCI and FSIQ on the facets of extraversion.

Predictor	Verbal Comprehension Index (VCI)				Full-Scale IQ (FSIQ)			
	B	SE B	*β*	*p*	B	SE B	*β*	*p*
Impulsivity (I)	-.359	.142	-.269	.013	-.244	.128	-.203	.058
Adventure Seeking (AS)	-.117	.150	-.086	.438	-.190	.135	-.156	.164
Detachment (D)	-.146	.132	-.117	.271	-.136	.119	-.121	.256

Adjusted R² = .088 for the VCI model and .079 for the FSIQ model. B = unstandardized regression coefficient; SE B = standard error of B; β = standardized regression coefficient.

### Within-group interaction patterns among personality traits

Thus far, the analyses have focused on associations between individual personality traits and cognitive outcomes within each age group. Because no significant one-dimensional associations were observed in the older group, exploratory analyses were conducted to examine whether configurations of personality traits were associated with cognitive functioning.

Extraversion (E), Neuroticism (N), and Aggressiveness (A) were dichotomized into high/low categories based on their respective mean values (E-high/low, N-high/low, and A-high/low, respectively). This procedure yielded eight possible personality configurations. These configurations were entered into a series of 2 * 2 * 2 ANOVAs with the cognitive measures as dependent variables. In the younger group, no significant main or interaction effects were observed. In the older group, however, significant interaction effects emerged. First, a significant Extraversion-by-Neuroticism interaction was observed for PSI, with higher PSI scores in the E-high/N-low subgroup than in the other subgroups (*F*(1, 84) = 4.542, *p* <.05, partial η²= .051). Second, a significant Extraversion-by-Neuroticism interaction was also observed for WMI, with higher WMI scores in the E-high/N-low subgroup than in the other subgroups (*F*(1, 84) = 5.284, *p* <.05, partial η²= .059).

These results indicate that, in older adults, cognitive functioning may be associated with configurations of personality traits rather than single traits in isolation.

### Facet-level characterization of interaction effects – older group

To further examine these interaction effects within the older group, participants with the E-high/N-low personality profile (Group 1; *N* = 35) were compared with the remaining participants (Group 2; *N* = 57). Facet-level differences were then analyzed using multivariate analysis of variance (MANOVA).

The facets of Extraversion included Impulsivity, Adventure Seeking, and Detachment. The facets of Neuroticism comprised Somatic Trait Anxiety, Psychic Trait Anxiety, Stress Susceptibility, Embitterment, and Lack of Assertiveness. Subsequently, one-way between-subjects MANOVAs were conducted for each personality trait, with the respective facets entered as dependent variables. Assumptions of homogeneity of variance-covariance matrices and equality of variance were confirmed, and moderate correlations were found among the dependent variables for both personality traits. A significant difference was observed between the two groups on the combined dependent variable Extraversion, *F*(3, 88) = 12.09, *p* <.001; Wilks’ Λ = .70; partial η² = .29. Follow-up univariate analyses using a Bonferroni-adjusted alpha level of.017 indicated that the groups differed significantly on two facets: Impulsivity, *F*(1, 90) = 16.203, *p* <.001, partial η² = .15, and Adventure Seeking, *F*(1, 90) = 26.909, *p* <.001, partial η² = .23. Mean scores for Group 1 were higher on both facets (*M* = 56.90 and *M* = 56.57, respectively) compared to Group 2 (*M* = 48.49 and *M* = 47.66, respectively). No other significant differences between the groups were found.

A significant difference was also observed between the two groups on the combined dependent variable Neuroticism, *F*(5, 86) = 5.188, *p* <.001, Wilks’ Λ = .80, partial η² = .23. Using a Bonferroni-adjusted alpha level of.01 follow-up analyses revealed significant group differences on two facets. First, Stress Susceptibility, *F*(1, 90) = 26.974, *p* <.001, partial η² = .23, with Group 1 scoring lower (*M* = 43.98) than Group 2 (*M* = 53.94). Second, Psychic Trait Anxiety, *F*(1, 90) = 10.404, *p* = .002, partial η² = .15, where Group 1 again scored lower (*M* = 44.01) compared to Group 2 (*M* = 49.23). No other significant differences between the groups were found.

These findings provide a more fine-grained understanding of how personality configurations are associated with cognitive performance within the older group.

### Summary of findings

Younger adults outperformed older adults on FSIQ, WMI, and PSI and exhibited higher Neuroticism. In the younger group, higher Extraversion was independently associated with lower FSIQ after adjustment for chronological age, sex, education, and the remaining personality traits. A nominal association between Extraversion and VCI did not remain significant after correction for multiple testing, although exploratory facet-level analyses implicated Impulsivity. In the older group, no significant one-dimensional personality-cognition associations were observed. However, exploratory analyses identified interaction-based patterns involving Extraversion and Neuroticism in relation to WMI and PSI. Overall, the findings suggest that personality-cognition associations differ across age groups, with a direct trait-level association in younger adults and more configurational patterns in older adults.

## Discussion

The aim of the present study was to examine the relationship between personality traits and cognitive functioning in the context of age, with a specific focus on whether these associations differ between younger and older adults and how they manifest within each age group. Overall, the findings indicate that personality–cognition associations differ not only in strength but also in structure across age groups. Whereas younger adults showed a direct association between Extraversion and global cognitive functioning, older adults displayed exploratory interaction-based patterns involving combinations of traits and their facets.

### Between-group differences in cognition and personality

With regard to between-group differences, younger adults outperformed older adults on Full-Scale IQ, working memory, and processing speed. Although the observed group differences are consistent with literature on age-related differences in working memory and processing speed (17, 20), the WAIS-IV index scores were age-normed. The findings therefore reflect differences relative to the respective age-based norms rather than unadjusted differences in raw cognitive performance and should not be interpreted as direct estimates of cognitive decline. In contrast, younger adults exhibited higher levels of Neuroticism than older adults, which accords with research suggesting that Neuroticism tends to decline across adulthood and that healthy older adults often show greater emotional stability (63, 64).

An interesting aspect of these between-group findings is that the younger group showed higher Neuroticism despite outperforming the older group cognitively. Although personality may contribute to individual variation in cognition (51, 65), the observed group differences likely reflect several interacting factors, including cohort characteristics, educational and experiential differences, and variation relative to the respective age-based WAIS-IV norms.

### Personality–cognition associations in younger adults

The within-group analyses revealed a distinct pattern among younger adults. After adjustment for chronological age, sex, years of education, and the remaining personality traits, higher Extraversion was uniquely associated with lower Full-Scale IQ. The association remained significant after adjustment for demographic variables, and the addition of personality traits accounted for a significant increase in explained variance.

A nominal association was also observed between Extraversion and Verbal Comprehension. However, this association did not remain significant after correction for multiple testing and should therefore be interpreted cautiously. It was nevertheless considered sufficiently informative to motivate subsequent exploratory facet-level analyses.

When Extraversion was decomposed into its constituent facets, the overall regression models were significant for both Full-Scale IQ and Verbal Comprehension. For Verbal Comprehension, Impulsivity emerged as the only significant facet-level predictor, whereas no individual facet uniquely predicted Full-Scale IQ. One possible interpretation is that the observed associations reflect differences in attentional control and self-regulation. Higher impulsivity may interfere with sustained concentration, careful reasoning, and the deliberate processing required for cognitively demanding tasks. This interpretation is consistent with previous work suggesting that individuals who are less behaviorally disinhibited may perform better in settings requiring focus, planning, and sustained mental effort (66).

Overall, the findings suggest a relatively direct association between Extraversion and global cognitive functioning in younger adults. The exploratory facet-level analyses provided some indication that impulsivity-related characteristics may contribute to the nominal association with Verbal Comprehension. However, no individual facet uniquely predicted Full-Scale IQ, suggesting that the association with global cognitive functioning may reflect the broader Extraversion construct or a combination of its lower-order components. These findings illustrate the potential value of combining trait- and facet-level approaches while underscoring the need for replication.

### Personality–cognition associations in older adults

The pattern in the older group differed markedly. No significant one-dimensional associations between personality traits and cognitive measures were observed. However, when combinations of traits were considered, a significant interaction emerged between Extraversion and Neuroticism. Older adults characterized by high Extraversion and low Neuroticism performed better on working memory and processing speed than the remaining older participants. However, the observed interaction effects were relatively small (partial η² = .051-.059), indicating that personality configurations explained only a limited proportion of the variance in cognitive functioning. In the present sample, the exploratory findings suggest that personality may relate to cognition less through isolated traits and more through trait configurations. Such a pattern is in line with the broader idea that the influence of one personality characteristic may depend on the level of another (60), and it indicates that a multidimensional perspective may be especially relevant in aging research.

A plausible interpretation of this interaction is that the combination of lower emotional vulnerability and higher behavioral engagement may be associated with better cognitive functioning among older adults. Low Neuroticism may reduce the disruptive effects of chronic worry, stress sensitivity, and self-doubt, whereas higher Extraversion may facilitate greater social, behavioral, and environmental engagement. Together, this combination may be associated with greater participation in stimulating activities, which in turn may be related to cognitive functioning. However, the direction of these associations cannot be determined from the present cross-sectional data. In this sense, the present findings are compatible with the view that cognitive aging is shaped not only by biological decline but also by the behavioral and emotional dispositions that influence how individuals interact with their environment.

The facet-level analyses in the older group deepen this interpretation. Specifically, compared with the remaining older adults, the cognitively better-performing subgroup scored lower on Stress Susceptibility and Psychic Trait Anxiety and higher on Impulsivity and Adventure Seeking. Stress Susceptibility refers to a tendency to become easily fatigued and to feel discomfort or unease when under pressure to act quickly. Psychic Trait Anxiety is characterized by frequent worrying, a tendency to anticipate potential problems, and a lack of self-confidence. Impulsivity is, as noted above, defined as acting on the spur of the moment, demonstrating a lack of planning, and exhibiting impulsive behavior. Adventure Seeking involves a preference for avoiding routine and a strong desire for change and action.

Notably, the facet-level analyses yielded moderate-to-large effect sizes (partial η² = .15-.23). While these findings should be interpreted cautiously and require replication, the magnitude of the effects suggests that the observed relationships may be of greater practical relevance than the trait-level associations.

More specifically, the observed pattern was associated not only with broad personality dimensions but also with narrower characteristics reflecting lower stress reactivity and greater behavioral initiative. One possible implication is that older adults who are less vulnerable to stress and more inclined toward action, novelty, and engagement may be more likely to maintain activity patterns that are cognitively beneficial. This interpretation aligns with Bielak and Gow (67), who emphasize that older adults who engage in more activities tend to perform better on cognitive tests and exhibit less cognitive decline with age. Similarly, Hultsch et al. (53) argue that participation in intellectually stimulating activities may serve as a buffer against cognitive decline. However, it is equally possible that better cognitive functioning facilitates greater engagement in such activities, or that both are influenced by other underlying factors.

An additional question raised by the present findings is why personality-cognition associations appeared to differ structurally between the age groups. In the younger adults, cognitive performance was related primarily to an individual trait, Extraversion, suggesting a relatively direct association between a specific behavioral tendency and cognitive functioning. In contrast, among older adults, cognitive performance was associated with a combination of personality characteristics, including both Extraversion- and Neuroticism-related facets. One possible explanation is that personality characteristics may become increasingly integrated over the lifespan through accumulated experiences and long-term behavioral patterns. In younger adulthood, individual traits may exert more direct effects on cognitive performance through mechanisms such as attentional control, self-regulation, and task engagement. In older adulthood, however, cognitive functioning may be influenced less by isolated traits and more by combinations of emotional, motivational, and behavioral dispositions that shape lifestyle, health behaviors, stress regulation, and participation in cognitively stimulating activities over many years.

From this perspective, the personality profile characterized by high Extraversion and low Neuroticism may not reflect the effect of any single trait but rather a broader pattern of behavioral engagement combined with emotional resilience. Such configurations may be particularly relevant in older adulthood because they influence how individuals respond to age-related challenges and maintain participation in social, intellectual, and everyday activities associated with cognitive functioning.

Taken together, these exploratory findings suggest that a multidimensional and facet-sensitive approach may be useful for understanding personality-cognition associations in older adulthood. Nevertheless, the cross-sectional nature of the study precludes conclusions regarding the directionality of these associations.

### Implications

The findings suggest that the structure of personality-cognition associations differs between younger and older adults. In younger adults, the relationship appears more direct and trait-specific, whereas in older adults it appears more configurational, involving interactions between traits and more differentiated facet-level patterns. This difference between the groups may help explain some of the inconsistency in previous research. Studies that examine only broad traits or only direct bivariate associations may overlook the possibility that personality–cognition links differ across age groups, with associations appearing more conditional and multidimensional among older adults. The present results also have potential practical implications. Although the cross-sectional design does not permit causal conclusions, the findings suggest that personality-related characteristics may be relevant when designing interventions to support cognitive functioning. In older adults, lower stress-related aspects of Neuroticism and higher engagement-related aspects of Extraversion were associated with better cognitive functioning, particularly in domains vulnerable to aging, such as working memory and processing speed. If replicated, these patterns may inform future research on whether interventions targeting stress regulation and active engagement could support cognitive functioning in older adulthood.

There is evidence that personality traits can be modified. For example, Stieger et al. (8) demonstrated that personality traits can be altered through digital interventions in nonclinical samples. However, whether such changes lead to improvements in cognitive functioning remains an open question. If future research supports the premise that personality changes may influence cognitive outcomes, one implication of the present findings is that future intervention research could examine whether changes in Neuroticism-related tendencies or adaptive aspects of Extraversion are associated with cognitive outcomes. Such approaches might, for instance, be informed by intervention frameworks proposed by Allemand and Flückiger (40), which have shown promising effects in previous studies (8).

Although speculative, this line of reasoning suggests that targeting personality-related processes may represent a potential avenue for supporting cognitive functioning in older age, particularly in domains such as working memory and processing speed that are susceptible to age-related decline (68, 69). In younger adults, the exploratory association between Impulsivity and lower Verbal Comprehension suggests that self-regulation and attentional control may warrant further investigation in educational or cognitively demanding contexts.

At the same time, these implications must be interpreted cautiously. The data do not allow conclusions about whether personality influences cognition, cognition influences personality, or whether both are shaped by shared underlying factors. Nevertheless, the results add to an emerging literature suggesting that personality is not necessarily fixed and that certain traits may be modifiable through psychological intervention.

### Limitations and strengths

Several limitations should be noted. The sample was based on convenience sampling and consisted of relatively high-functioning individuals, which may limit the generalizability of the findings. The cross-sectional design precludes conclusions regarding developmental change or causal relationships. Information on depressive symptoms, anxiety, medical conditions, and medication use was unavailable and could therefore not be included in the analyses. Although participants were screened for relevant medical and psychiatric conditions, no formal medical examination was conducted, and undetected clinical or preclinical conditions cannot be ruled out. In addition, personality traits were dichotomized in the exploratory personality-configuration analyses. While this decision was guided by the study aims, it may have reduced statistical power and obscured potentially informative variation within groups. Future studies should examine personality configurations using continuous measures and interaction-based approaches. Furthermore, the large number of statistical tests conducted increases the risk of Type I error. Years of education was missing for 22 participants, and the covariate-adjusted regression analyses were therefore based on complete cases. The reduced analytical samples may have limited statistical power and could have introduced bias if the missingness was systematic. The exploratory personality-configuration analyses were not adjusted for demographic covariates and should therefore be interpreted cautiously. In addition, no *a priori* power analysis was conducted, and the subgroup and interaction analyses were based on relatively small cell sizes, potentially limiting statistical power and the stability of the observed effects. Effect sizes varied across analyses: the incremental contribution of personality traits to FSIQ in younger adults was moderate, whereas the interaction effects in older adults were small. The practical significance of these findings should therefore be interpreted cautiously until replicated.

The use of the SSP, although informative and psychometrically sound, constrains comparability with studies based on the Five-Factor Model and does not include an explicit measure of Openness, which has often shown robust associations with cognition in previous research. Additionally, the observed associations may reflect several alternative mechanisms, including reverse causality or common underlying factors influencing both personality and cognitive functioning. Longitudinal studies are needed to clarify the direction of these relationships.

Despite these limitations, the study has notable strengths. The use of a comprehensive neuropsychological test battery allowed for the assessment of multiple cognitive domains, and the combination of trait-level, facet-level, and interaction-based analyses provided a more differentiated picture of personality–cognition associations than would have been possible with broad trait measures alone. This multi-level approach may be particularly useful in addressing contradictory findings in the literature.

## Conclusion

The present study suggests that personality-cognition associations differ across age groups not only in magnitude but also in structure. In younger adults, higher Extraversion showed a direct negative association with global cognitive functioning after adjustment for demographic factors and the remaining personality traits. In older adults, no significant one-dimensional associations were observed, but exploratory analyses identified configurations of Extraversion- and Neuroticism-related characteristics associated with working memory and processing speed. These findings underscore the value of examining both individual traits and trait configurations while distinguishing robust findings from exploratory facet-level patterns.

A potentially valuable direction for future research is the examination of the combined effects of personality traits on cognitive ability to identify more detailed personality profiles and to gain a deeper understanding of the within-individual structure of personality, particularly among older adults. A person-centered approach to personality focuses on the organization of traits within an individual (70), allowing for the classification of individuals into personality types based on similar trait configurations (71). Combining a person-centered approach with a facet-level perspective may provide a more nuanced understanding of how specific components of personality interact with different cognitive domains.

Such specificity may help address inconsistencies observed across studies, where divergent findings have often been attributed to methodological differences or overly coarse trait-level analyses. In sum, adopting a more fine-grained perspective on personality and cognition holds considerable promise for advancing theoretical models and improving practical applications in this field.

## Data Availability

The raw data supporting the conclusions of this article will be made available by the authors, without undue reservation.
